# White-Matter Hyperintensity Load and Differences in Resting-State Network Connectivity Based on Mild Cognitive Impairment Subtype

**DOI:** 10.3389/fnagi.2021.737359

**Published:** 2021-10-07

**Authors:** Martina Vettore, Matteo De Marco, Claudia Pallucca, Matteo Bendini, Maurizio Gallucci, Annalena Venneri

**Affiliations:** ^1^Department of Neuroscience, University of Sheffield, Sheffield, United Kingdom; ^2^Department of General Psychology, University of Padua, Padua, Italy; ^3^Department of Life Sciences, Brunel University London, Uxbridge, United Kingdom; ^4^Department of Clinical Neurosciences, University of Cambridge, Cambridge, United Kingdom; ^5^Cognitive Impairment Center, Local Health Authority n.2 Marca Trevigiana, Treviso, Italy; ^6^Unit of Neuroradiology, Treviso Regional Hospital, Treviso, Italy

**Keywords:** dementia, Alzheimer’s disease, haemodynamic, small vessel disease, independent component analysis

## Abstract

“Mild cognitive impairment” (MCI) is a diagnosis characterised by deficits in episodic memory (aMCI) or in other non-memory domains (naMCI). Although the definition of subtypes is helpful in clinical classification, it provides little insight on the variability of neurofunctional mechanisms (i.e., resting-state brain networks) at the basis of symptoms. In particular, it is unknown whether the presence of a high load of white-matter hyperintensities (WMHs) has a comparable effect on these functional networks in aMCI and naMCI patients. This question was addressed in a cohort of 123 MCI patients who had completed an MRI protocol inclusive of T1-weighted, fluid-attenuated inversion recovery (FLAIR) and resting-state fMRI sequences. T1-weighted and FLAIR images were processed with the Lesion Segmentation Toolbox to quantify whole-brain WMH volumes. The CONN toolbox was used to preprocess all fMRI images and to run an independent component analysis for the identification of four large-scale haemodynamic networks of cognitive relevance (i.e., default-mode, salience, left frontoparietal, and right frontoparietal networks) and one control network (i.e., visual network). Patients were classified based on MCI subtype (i.e., aMCI vs. naMCI) and WMH burden (i.e., low vs. high). Maps of large-scale networks were then modelled as a function of the MCI subtype-by-WMH burden interaction. Beyond the main effects of MCI subtype and WMH burden, a significant interaction was found in the salience and left frontoparietal networks. Having a low WMH burden was significantly more associated with stronger salience-network connectivity in aMCI (than in naMCI) in the right insula, and with stronger left frontoparietal-network connectivity in the right frontoinsular cortex. Vice versa, having a low WMH burden was significantly more associated with left-frontoparietal network connectivity in naMCI (than in aMCI) in the left mediotemporal lobe. The association between WMH burden and strength of connectivity of resting-state functional networks differs between aMCI and naMCI patients. Although exploratory in nature, these findings indicate that clinical profiles reflect mechanistic interactions that may play a central role in the definition of diagnostic and prognostic statuses.

## Introduction

Mild cognitive impairment (MCI) is a diagnostic entity used to describe a disorder characterised by mild deficits in cognition that do not usually interfere with a patient’s ability to lead an independent life. When first introduced, MCI described an intermediate stage between normal ageing and Alzheimer’s disease (AD) dementia, and impairment in memory was required for the diagnosis to be made (Petersen et al., 1999). In time, the concept of MCI has been expanded to include cognitive impairments outside the memory domain and has become applicable to aetiologies other than AD (Winblad et al., 2004). Classification of MCI in different subtypes was introduced to recognise and distinguish among probable underlying aetiologies. A first classification is based on the impaired cognitive domain(s), with patients classified as “amnestic” MCI (aMCI) if their performance on tests of episodic memory is poor (usually 1–1.5 SD below the mean of age and education-matched peers), or, otherwise, as “non-amnestic” MCI (naMCI) for deficits in any other domains. This classification can be helpful to clinicians since aMCI is most commonly observed in patients who later progress to typical AD dementia, whereas naMCI is most likely associated with other, non-AD forms of dementia, such as dementia with Lewy bodies or frontotemporal dementia (Petersen, 2004; Yaffe et al., 2006; Albert et al., 2011; Ferman et al., 2013).

White-matter hyperintensities (WMHs), a neuroimaging feature associated with small-vessel disease, are a common finding in MCI patients, and do not seem to vary in severity across subtypes, at least on visual inspection (van de Pol et al., 2009; Boyle et al., 2016). WMHs are typically evidenced as areas of increased signal intensity in T2-weighted, proton-density and fluid-attenuated inversion recovery (FLAIR) MRI images. WMHs have been associated with a higher risk of developing MCI, vascular dementia and AD dementia, and overall appear to promote a faster decline in global cognitive performance (Debette and Markus, 2010; Boyle et al., 2016).

Experimental evidence suggests that small-vessel disease interacts with the diagnosis-defining pathological features of AD (i.e., amyloid plaques and neurofibrillary tangles). Both amyloid and TAU pathology, in fact, appear to affect the integrity of small vessels (Greenberg et al., 2020; Michalicova et al., 2020). In addition, a number of studies indicates that WMH burden may also contribute to neural pathophysiological changes, either in an additive or synergic way, by lowering the threshold for the clinical manifestation of dementia, or by being mechanistically linked to the core underlying pathophysiological processes (Breteler, 2000; Brickman et al., 2009; Gorelick et al., 2011; Kalaria and Ihara, 2013). On this note, a number of studies found a statistical association between WMH burden and amyloid β depositions (Brickman et al., 2015; Zhou et al., 2015; for a review, see Roseborough et al., 2017).

Recent studies have reported that WMH burden appears to disrupt the integrity of brain functional networks of healthy controls and MCI patients. Decreased functional connectivity within the default mode network (DMN) has been reported in association with WMH burden both in healthy adults (Reijmer et al., 2015; Liu et al., 2019) and in adults with cognitive impairment (Zhou et al., 2015; Liu et al., 2019). Positive associations between WMH burden and connectivity, on the other hand, have been found in the salience network (SN), suggesting a compensation mechanism (Cheng et al., 2017; De Marco et al., 2017b). The SN is involved in the detection and integration of endogenous and exogenous stimuli relevant for behaviour (Seeley et al., 2007; Menon, 2015) and a positive association would translate into the necessity of relying on more neural resources during the phase of stimulus selection and integration. There is also evidence linking WMH burden to executive dysfunction (Bombois et al., 2007; Debette and Markus, 2010), and Jacobs et al. (2012) studied the effect of WMH burden on functional networks involved in executive functions in MCI patients. WMHs affecting the territory underlying the frontoparietal and frontal–parietal-subcortical networks were indeed associated with decline in executive functions in MCI patients.

Most studies investigating associations of WMH burden with connectivity of resting state networks have considered MCI as a unique group, without any distinction of subtype. Since aMCI and naMCI present different clinical features and might have a different progression rate, they may originate from disruption of different underlying neural mechanisms and neuropathological processes. Distinct aetiologies may, therefore, interact differently with WMH burden to foster changes in functional connectivity, eventually determining the different clinical phenotypes.

In this study, we divided MCI patients into four groups based on WMH burden (high/low) and MCI subtype (aMCI/naMCI) to investigate how activity within major resting-state brain networks of cognitive relevance is modulated by the interaction of these two variables. We expected that WMH burden would be a statistically significant predictor of network connectivity, and that differences in network expression among subgroups would be reflective of patients’ symptoms. In particular, we anticipated that effects in regions deputed to memory processing would be particularly relevant in aMCI patients, while effects in frontal and parietal regions would be relevant in naMCI patients, as this group typically shows a clinical profile characterised by executive or visuospatial impairment. Since this is, to our knowledge, the first study to investigate this interaction, we adopted an exploratory approach and analysed maps of connectivity voxel by voxel.

## Materials and Methods

### Participants

This study included 123 patients with a clinical diagnosis of MCI enrolled as part of two initiatives: the Sheffield Ageing Database (United Kingdom) and the TREviso DEMentia (TREDEM) study (Italy) (Gallucci et al., 2012, 2018). Recruitment procedures were completed in the context of tertiary-care clinical facilities for memory disorders, where a diagnosis of MCI due to AD was formulated for each patient on the basis of published clinical criteria (Albert et al., 2011), and was confirmed by a consensus of clinicians, inclusive of a senior neurologist, a neuropsychologist and a neuroradiologist. Each patient underwent a brain MRI and an extensive neuropsychological assessment. Exclusion criteria were defined as follows: clinical conditions of concern other than reduction in mental abilities, significant disabilities or psychiatric symptoms of concern, evidence of MRI abnormalities different from those expected in prodromal AD, pharmacological treatments with cholinergic drugs, psychotropic medications, drugs with toxic effects to internal organs or taken for research purposes, uncontrolled seizures (presence/diagnosis), sick sinus syndrome, peptic ulcer, neuropathy with conduction difficulties, abnormal levels of folates, vitamin B12 or thyroid stimulating hormone. Patients who did not undergo a full neuropsychological examination and that did not have structural and functional scans were also not considered for inclusion.

### MRI Acquisition and Modelling

A harmonised protocol of anatomical and resting-state functional MRI images was devised as part of the study using 1.5 T systems (Philips Achieva and Siemens Avanto for patients recruited as part of the Sheffield Ageing Database and TREDEM initiatives, respectively). Anatomical images included three-dimensional T1-weighted and T2-weighted FLAIR sequences. Specifications for each sequence are detailed in Table 1.

**TABLE 1:** Technical specifications of the MRI sequences used in this study.

	Sheffield ageing database (*n* = 85)	TREDEM (*n* = 38)
**T1-weighted**		
Voxel size (mm^3^)	1.1 × 1.1 × 0.6	1.0 × 1.0 × 1.0
Repetition time (ms)	7.42	9.50
Echo delay time (ms)	3.42	4.76
Matrix size	256 × 256	512 × 512
Number of slices	280	160
**FLAIR**		
Voxel size (mm^3^)	0.75 × 0.75 × 4.93	0.45 × 0.45 × 6.50
Repetition time (ms)	8000	9000
Echo delay time (ms)	125	92
Matrix size	320 × 320	392 × 512
Number of slices	30	27
**Resting-state fMRI**		
Voxel size (mm^3^)	2.87 × 2.87 × 6.00	3.81 × 3.81 × 5.00
Repetition time (ms)	2000	2000
Echo delay time (ms)	50	40
Matrix size	64 × 64	64 × 64
Field of view (mm)	230	244
Slices per volume	20	28

*TREDEM, TREviso DEMentia study.*

Ten dummy volumes (20 s) were initially set up to allow the scanning environment to reach electromagnetic equilibrium prior to resting-state acquisitions. For these scans, patients were instructed to lay as still as possible with their eyes closed and in-scanner motion was minimised adapting the coil with foam padding to limit movement of the head.

The entire processing of MRI images was carried out with Matlab R2014a (Mathworks Inc., United Kingdom) and Statistical Parametric Mapping (SPM) 12 (Wellcome Centre for Human Neuroimaging, London, United Kingdom). T1-weighted scans were initially segmented to obtain native-space tissue-class maps of grey matter, white matter, and cerebrospinal fluid (Ashburner and Friston, 2005). The ‘‘get_totals’’ function^1^ was then used to quantify each tissue-class map and thus obtain individual total intracranial volumes (TIVs).

T1-weighted and FLAIR scans were used to calculate WMH volumes *via* the Lesion Segmentation Tool toolbox (Schmidt et al., 2012; Birdsill et al., 2014). The Lesion Growth Algorithm (LGA) function was applied at a threshold of κ = 0.3 and the resulting individual WMH volume, expressed in ml, was divided by its respective TIV to obtain a fractional index of white matter lesion volume. A κ = 0.3 has been indicated as the best performing threshold by an earlier methodological study (Schmidt et al., 2012) and has been used in previous research as the optimal value to separate WMH from normal tissue (De Marco et al., 2017b; Bentham et al., 2019; Manca et al., 2021).

Resting state functional MRI scans were preprocessed and modelled using SPM and the CONN platform toolbox (Whitfield-Gabrieli and Nieto-Castanon, 2012). A first realignment was carried out using SPM to visualise and assess in-scanner motion plots for initial quality-check purposes. At this preliminary stage, scans with movement parameters in the range of ±3 mm and ±3° were considered acceptable. As a result, one scan was flagged up as problematic and was thus shortened to 135 consecutive volumes. All remaining scans included 240 volumes (equal to an 8-min acquisition) for all patients of the Sheffield Ageing Database, or 180 volumes (equal to a 6-min acquisition) for all patients recruited as part of the TREDEM initiative. Preprocessing was then restarted *via* CONN. Images were slice-timed, realigned, coregistered to their respective T1-weighted anatomical image, normalised to the Montreal Neurological Institute space template and smoothed with a 6-mm Gaussian kernel. A range of denoising methods was then applied to minimise the impact of artefactual sources of signal variability. This included band-pass filtering (0.01–0.1 Hz), scrubbing (volumes showing displacement larger than the 97th percentile were censored), regressing out of the first 10 principal components (aCompCor) calculated within the maps of white matter (5 components) and cerebrospinal fluid (5 components) and regressing out of 24 head motion parameters, including linear and rotational indices, their temporal derivatives and their squared values.

**FIGURE 1:**
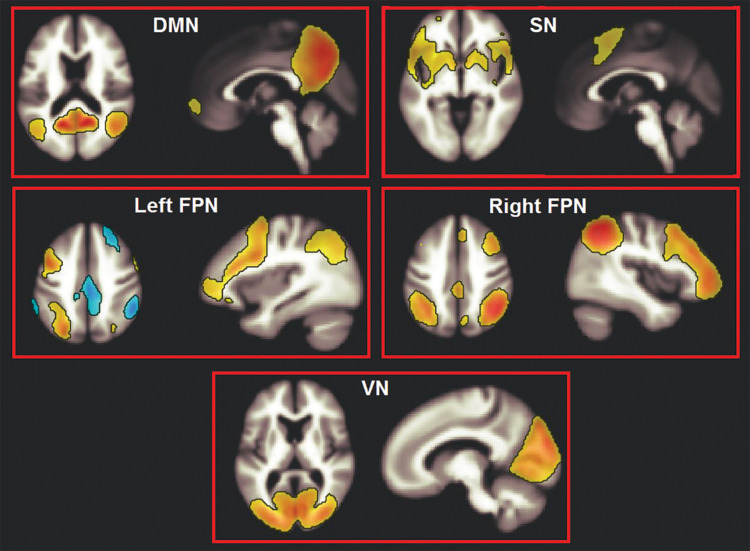
The five large-scale haemodynamic networks investigated in this study. DMN, default mode network; SN, salience network; FPN, frontoparietal network; VN, visual network.

An independent component analysis (ICA) was run to obtain maps of network connectivity (Calhoun et al., 2001). This is a data-driven statistical analysis that assumes that independent sources of signal combine linearly and result in the observed data (Rosazza et al., 2012). The Fast-ICA reduction principle was used and 20 components were computed, in line with previously published research (Biswal et al., 2010). The spatial contour of the resulting components was inspected, four of which, spatially consistent with networks of interest, were selected. These networks (DMN, SN, and the left and right frontoparietal networks) play a major role in supporting cognitive functioning. A fifth, non-cognitive network (the occipital visual network) was then selected as control component (Figure 1).

### Data Modelling

Two statistical predictors of interest were defined to address the experimental hypothesis: WMH load and MCI subtype. A median split was carried out to separate normalised WMH volumes into “low WMH load” and “high WMH load”. To define MCI subtypes, patients were classified as aMCI or naMCI based on their cognitive profile, i.e., patients were labelled as “aMCI” when impaired performance was recorded on at least one test of episodic memory, i.e., Rey-Osterrieth Complex Figure delayed recall, Prose Memory and Rey Auditory Verbal Learning Test (specific tests were used at each recruitment centre). Sub-sample numerosity distributed as follows: aMCI-low WMH load: *n* = 43; naMCI-low WMH load: *n* = 19; aMCI-high WMH load: *n* = 42; naMCI-high WMH load: *n* = 19.

The interaction between WMH load and MCI subtype was then modelled for each network of interest. Centre of recruitment, age, education level (in years), TIV, and global cognitive levels (i.e., Mini Mental State Examination score) were added as covariates. TIV also served to control for sex differences, since these two variables are strongly associated (Pintzka et al., 2015). Significance level was set at a cluster-forming threshold equal to *p* < 0.001 (uncorrected) and clusters surviving a cluster-level FWE-corrected threshold of *p* < 0.05 were reported as significant. Result coordinates were converted from Montreal Neurological Institute space into Talairach space and interpreted using the Talairach Daemon Client (Lancaster et al., 2000). To simplify the interpretation of the interaction contrasts, these were operationalised in terms of ‘‘low-WMH advantage’’ being larger among aMCI or naMCI. G^∗^Power (version 3.1.9.7^2^) was used to estimate statistical power and ensure that our available sample size was sufficient to detect a significant statistical effect. With standard type I error and statistical power probabilities (5% and 80%, respectively), four groups and four covariates, a cohort size of *n* = 123 participants was sufficiently large to detect a medium effect size as significant (*f* = 0.255).

## Results

**FIGURE 2:**
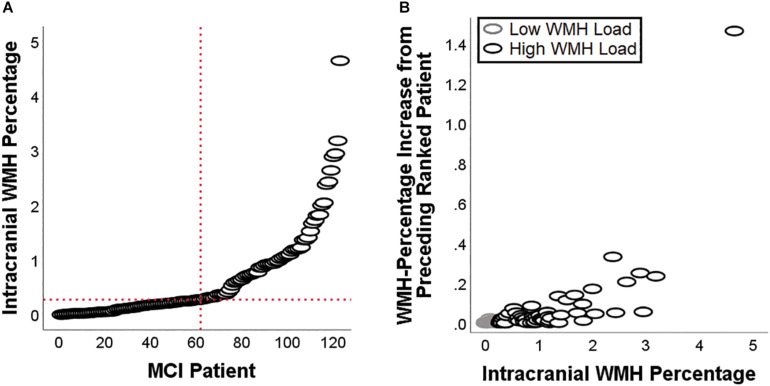
White-matter hyperintensities load for each of the patients included in this study **(A)**, and patient-to-patient increase in WMH load **(B)**. The latter scatterplot highlights the difference in dispersion between patients with a low load and patients with a high load.

**FIGURE 3:**
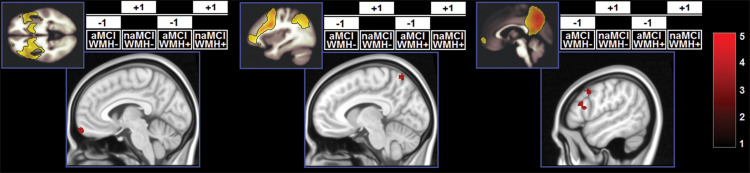
Clusters in which a significant main effect of WMH load was found. *z*-Scores are indicated by the colour bar. aMCI, amnestic MCI; naMCI, non-amnestic MCI; WMH–, low WMH; WMH+, high WMH.

**FIGURE 4:**
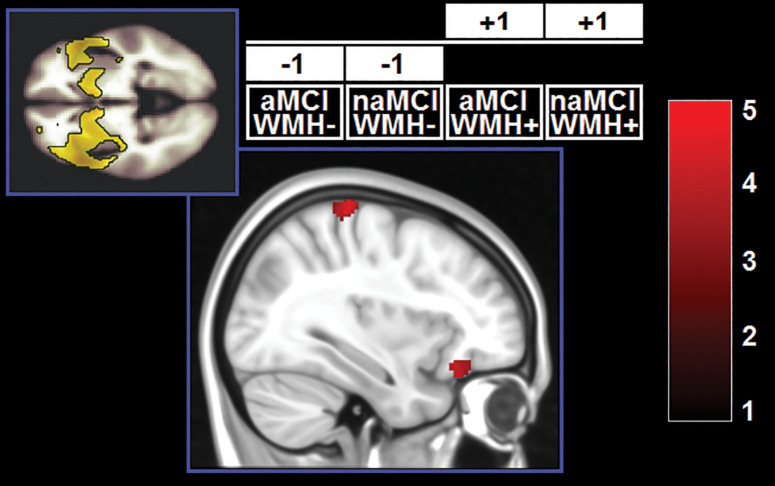
Clusters in which a significant main effect of subtype was found. *z*-Scores are indicated by the colour bar. aMCI, amnestic MCI; naMCI, non-amnestic MCI; WMH–, low WMH; WMH+, high WMH.

**FIGURE 5:**
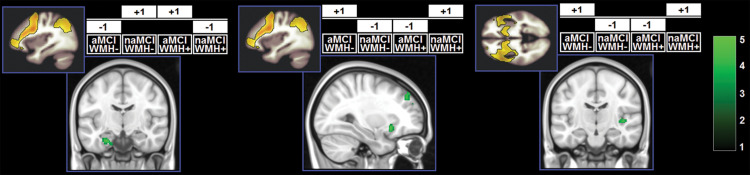
Clusters in which a significant interaction was found. *z*-Scores are indicated by the colour bar. Specific contrasts are shown for each model associated with a significant findings. aMCI, amnestic MCI; naMCI, non-amnestic MCI; WMH–, low WMH; WMH+, high WMH.

Demographic and neuropsychological characteristics of the groups are reported in Table 2. The distribution of individual WMH loads is shown in Figure 2. The median split separated the cohort into a homogeneous group of patients with low load and very low dispersion (*s*^2^ = 0.008) and a group of patients with a load larger than 0.28% of their intracranial space and a significantly larger dispersion (*s*^2^ = 0.716, Levene test’s *p* = 7.78^–11^). All significant main effects and interactions are listed in Table 3 and illustrated in Figures 3–5. Patients with a high WMH load had more SN connectivity in right pericentral areas and in the right inferior frontal lobe (Figure 3). naMCI patients had more SN connectivity in the medial frontal gyrus, more DMN connectivity in the left dorsolateral prefrontal cortex and more left FPN connectivity in the precuneus (Figure 4).

**TABLE 2:** Sample characterisation.

	aMCI		naMCI		*p* (ANOVA)
	Low WMH load	High WMH load	Low WMH load	High WMH load	
**Demographic variables**					
Age (years)	71.40 (6.40)	76.83 (5.85)	71.05 (5.82)	75.74 (5.30)	WMH (*p* < 0.001)
Education (years)	9.74 (3.80)	10.14 (4.43)	9.42 (4.15)	11.11 (3.93)	n.s.
Sex (f/m)	19/24	25/17	9/10	12/7	n.s.
**Neurovolumetric variables**					
TIV (ml)	1399.78 (152.94)	1454.49 (4.43)	1388.38 (171.74)	1488.98 (130.59)	WMH (*p* = 0.008)
WMH load fraction	0.0013 (0.0009)	0.0124 (0.0092)	0.0013 (0.0009)	0.0095 (0.0064)	WMH (*p* < 0.001)
**Neurocognitive variables**					
MMSE	27.48 (1.82)	26.52 (2.36)	27.11 (2.16)	27.89 (1.45)	Subtype-by-WMH (*p* = 0.031)
Digit cancellation test	46.23 (7.95)	44.76 (8.36)	47.78 (5.67)	50.63 (6.31)	Subtype (*p* = 0.014)
Letter fluency test	26.23 (9.53)	25.52 (11.85)	28.17 (12.72)	28.79 (10.62)	n.s.
Token test	32.17 (3.14)	31.57 (3.64)	31.58 (2.97)	32.21 (2.85)	n.s.
Immediate recall*	0.281 (0.157)	0.239 (0.105)	0.414 (0.106)	0.339 (0.100)	Subtype (*p* < 0.001); WMH (*p* = 0.019)
Delayed recall*	0.253 (0.186)	0.195 (0.157)	0.453 (0.130)	0.404 (0.137)	Subtype (*p* < 0.001)

aMCI, amnestic mild cognitive impairment; MMSE, Mini-Mental State Examination; naMCI, non-amnestic mild cognitive impairment; TIV, total intracranial volume; WMHs, white-matter hyperintensities. *Variable expressed as a proportion of total score to harmonise scores obtained in different tests of memory between the two cohorts.

**TABLE 3:** Main effects and interactions emerged from the analyses.

Brain Region	Side	Cluster Size (voxels)	Cluster-Level *pFWE*	z Score	Talairach Coordinates		
					*x*	*y*	*z*
**Main Effects**							
*High WMH Load > Low WMH Load – Salience Network*							
Precentral Gyrus (BA4)	R	110	0.020	4.58	36	−26	64
Postcentral Gyrus (BA2)	R			3.41	30	−35	68
Inferior Frontal Gyrus (BA47)	R	102	0.029	4.04	38	26	−18
*Non-Amnestic > Amnestic – Salience Network*							
Medial Frontal Gyrus (BA11)	L	134	0.007	4.63	−10	63	−15
*Non-Amnestic > Amnestic – Default-Mode Network*							
Middle Frontal Gyrus (BA8)	L	99	0.026	4.33	−48	12	38
Inferior Frontal Gyrus (BA45)	L	151	0.002	3.89	−57	11	18
Inferior Frontal Gyrus (BA45)	L			3.79	−48	16	14
Inferior Frontal Gyrus (BA44)	L			3.74	−57	16	12
*Non-Amnestic > Amnestic – Left Frontoparietal Network*							
Precuneus (BA7)	L	94	0.028	4.20	−12	−50	54
Precuneus (BA7)	L			3.83	−6	−57	62
Precuneus (BA7)	L			3.18	−20	−46	56
**Interaction Effects**							
*Low WMH Load Advantage Stronger in Amnestic –*							
* Salience Network*							
Insula (BA13)	R	109	0.021	4.16	42	−16	1
Insula (BA13)	R			3.76	38	−25	3
*Low WMH Load Advantage Stronger in Amnestic –*							
* Left Frontoparietal Network*							
Insula (BA13)	R	131	0.008	4.57	30	17	−8
Middle Frontal Gyrus (BA9)	R			4.43	28	42	33
Superior Frontal Gyrus (BA9)	R			3.84	24	54	32
Superior Frontal Gyrus (BA10)	R			3.66	26	54	23
*Low WMH Load Advantage Stronger in Non-Amnestic –*							
* Left Frontoparietal Network*							
Parahippocampal Gyrus (BA35)	L	124	0.011	4.29	36	−26	64
Uncus	L			1.03	30	−35	68
Parahippocampal Gyrus (BA35)	L			3.65	38	26	−18

*BA, Brodmann area; FWE, family-wise error; L, left; R, right; WMHs, white-matter hyperintensities.*

A significant effect of the WMH-by-subtype interaction was found in the SN and left FPN (Figure 5). The low-WMH advantage was significantly stronger at the core of the SN (right insula) among aMCI patients. Similarly, aMCI patients showed a more significant low-WMH advantage within the left FPN, in a right fronto-insular cluster extending to the right insula and right dorsolateral prefrontal territory. Conversely, naMCI patients showed a more significant low-WMH advantage within the left FPN in the left mediotemporal lobe.

## Discussion

Mild cognitive impairment is a clinical diagnosis that may be associated with a variety of neurodegenerative processes (Winblad et al., 2004). Although the prodromal phase of AD is more typically characterised by deficits in episodic memory, any MCI subtype may convert to dementia of the AD type (Fischer et al., 2007). It is, thus, clinically important to characterise the complexity of the mechanisms that are responsible for neurofunctional alterations in abnormal ageing. Since WMHs are common findings in older adults, in this study we investigated whether a low/high WMH load has a differential impact on resting-state circuitry in a way that depends on MCI subtype.

The effect of the WMH-by-subtype interaction was detectable in two large-scale pathways: the SN and left FPN. In the models analysing SN function, the “low-WMH load advantage” was significantly larger among aMCI patients in the right insula. This indicates that WMH burden plays a more central role in the expression of the SN when the profile is of the amnestic type. Integrity of the SN supports memory performance (Andreano et al., 2017), but studies characterising the SN profile in aMCI have led to conflicting findings, with some studies indicating reduction (He et al., 2014) and others reporting increases (Li et al., 2020) in SN expression. Although we found significant main effects of “subtype” and “WMH load”, indicating that aMCI patients and patients with a low WMH load have reduced regional expression of the SN, these were superseded by a significant effect of the interaction. These findings indicate, therefore, that changes in functional connectivity of the SN may be driven by an interplay of mechanisms, and that WMH load may contribute to this interaction. This principle may play a central role at a clinical level, since neurofunctional alterations are at the basis of cognitive dysfunction. More specifically, from the analyses of the SN it was amnestic patients who were particularly susceptible to the detrimental effects of WMHs. Although episodic memory is supported by a wide-distributed large-scale network, its efficiency is dependent on the integrity of the hippocampus (Dickerson and Eichenbaum, 2010). On the other hand, the functions impaired in MCI patients with dysexecutive or visuospatial deficits are typically sustained by networks less homogeneously dependent on a single structure. We argue that this is the main reason why variability in WMHs has a smaller statistical impact among naMCI patients. Vice versa, aMCI patients whose white-matter tracts are relatively spared by WMH pathology can benefit more from higher level of SN neurofunctional integrity.

An interaction effect in the same direction also emerged from the analysis of the left FPN. Right frontoinsular regions are the core of SN processing and are responsible for transitional switching between DMN and FPN (Sridharan et al., 2008). Increased FPN expression in this region indicates tighter coupling between SN and FPN. Loss of inter-network functional connectivity between the right frontoinsular cortex accompanies cognitive decline in MCI patients (He et al., 2014). Along this interpretational line, we found that in patients with aMCI a low-WMH burden appears to be advantageous as it results in a tighter SN-FPN inter-network connectivity. Within the left FPN, however, an effect in the opposite direction was also found. Having a low-WMH load was associated with stronger network expression in the left mediotemporal lobe. This territory shows distinctive regional atrophy at the MCI stage of AD (Ferreira et al., 2011) and is, thus, particularly dysfunctional in those MCI patients who have underlying AD pathology. We argue that patients with naMCI benefit the most from a low-WMH burden because, as shown by their performance on episodic memory tests, their mediotemporal functioning is within normal levels, and, therefore, a “typical” effect of WMH load can be detected. Vice versa, WMH load would have little or no effect in patients with memory impairment due to dysfunctional mediotemporal areas.

No significant effect was found for the DMN. This expands the findings provided by previous exploratory studies carried out in small samples (*n* < 50) of patients with cognitive impairment (Zhou et al., 2015; Liu et al., 2019). The DMN is a neurofunctional system characterised by a degree of vulnerability to multiple processes, including ageing and AD pathology (Jagust, 2013). A recent meta-analysis, however, found considerable variability in the pattern of DMN alterations seen in patients with MCI (Eyler et al., 2019). Such heterogeneity suggests that there is no direct and clear-cut link between network dysfunction and a clinical diagnosis of MCI and that this link is likely to be influenced by various mechanisms and intervenient factors, such as neural compensation and reserve (Stern, 2009) that are at the basis of inter-individual differences in the functional circuitry that supports cognitive functioning. It has also been proposed that the pattern of regional atrophy typically seen in ageing may act as a promoter of functional plasticity, because task activation in older adults tends to increase in areas that are subjected to age-associated volumetric decrease (Greenwood, 2007). A recent study confirmed this negative association between regional structure and network expression in healthy adults, and also found inverse (positive) associations in patients with AD, indicating that those with reduced atrophy were also those with a stronger network expression (Sarli et al., 2021). Within this context, WMHs are a further contributor to the reorganisation of the neurofunctional architecture, as reported by a number of investigations (Reijmer et al., 2015; Zhou et al., 2015; Cheng et al., 2017; De Marco et al., 2017b; Liu et al., 2019). The resulting picture is, thus, that of large-scale networks being under the effect of multiple concurrent (and, plausibly, interactive) processes. This study adds to this body of literature, providing evidence that significant interactions exist between WMH load and the expression of the neurofunctional architecture underlying distinct cognitive profiles. It is also worth noting that, while the four subgroups included in this study were, on average, between 71 and 76 years old, MCI is a condition that may be diagnosed at any point of middle-to-old adulthood. Since ageing is associated with increasing WMH load as well as physiological changes in network expression (and with a number of other neurological processes such as cortical atrophy and hypometabolism), it still needs to be confirmed whether the associations described in this study will hold valid at other stages of ageing. Also, since network alterations at the MCI stage may manifest either as a reduction or as an over-expression of neurofunctional pathways, plausibly the outcome of either compensatory and/or maladaptive processes (Gardini et al., 2015; De Marco et al., 2017a), it is important to be cautious when interpreting findings that are expressed as unidirectional (i.e., in the form of positive or negative associations) statistical effects.

There are two limitations to this study to take into consideration. First, we adopted a methodology that computes a global load of WMHs that does not allow a precise localisation within different brain regions (i.e., deep vs. periventricular white matter). Although this is a methodological aspect of potential improvement to investigate the impact of WMHs on neurofunctional pathways in the future, it is known that the frontal lobe seems to be particularly susceptible to this type of damage, regardless of where hyperintensities are located (Tullberg et al., 2004). This indicates that the calculation of a global WMH load measure is of interest, since the effect of this type of damage seems to converge toward a common target. The two groups of patients resulting from the median split showed different distributions, with patients with a low WMH load distributing homogeneously in proximity to 0 (ranging from 0 to 0.276% of intracranial space) and patients with a high-WMH load showing a significantly larger dispersion (ranging from 0.285 to 4.649% of intracranial space). In addition, a binary classification of patients (i.e., as having a “low”-vs.-“high” WMH load) is of clinical relevance, since this information is usually taken into account by clinicians to define the most adequate approach to treatment. In this respect, binarising the load of hyperintensities facilitates the operationalisation of the interaction term and helps draw clearer interpretations that are of immediate clinical value. Second, we did not separate our cohort of MCI patients into “single-domain” and “multiple-domain”. This further subdivision would require larger cohorts and would also require a more extensive number of cognitive tests in order to characterise each domain in equal detail. Finally, although not a limitation, we acknowledge that additional or different correction factors might have been included in the models. Given that we corrected for those factors that account for the greatest influence on brain parameters, it is unlikely, however, that this might have led to substantial differences in the pattern of findings.

In conclusion, our study explored for the first time the interaction of WMH burden and MCI subtype. This topic will become increasingly more relevant as the contribution of vascular damage to cognitive deficits across different types of dementia outside the vascular type is being recognised. With the need of identifying individuals at risk for dementia earlier on, it is important to identify all the variables that can contribute to their cognitive phenotype and investigate how the interaction among these variables is reflected at the pathophysiological level.

## Data Availability Statement

The raw data supporting the conclusions of this article will be made available by the authors, without undue reservation.

## Ethics Statement

Ethical review and approval was not required for the study on human participants in accordance with the local legislation and institutional requirements. The patients/participants provided their written informed consent to participate in this study.

## Author Contributions

MV carried out the literature search, ran the analyses, interpreted the findings, and wrote the manuscript. MD contributed to data collection, designed this study, ran the analyses, and interpreted the findings. CP and MB contributed to data collection and reviewed the manuscript. MG coordinated the data collection and reviewed the manuscript. AV coordinated the data collection, conceived this study, reviewed and finalised the manuscript. All authors contributed to the article and approved the submitted version.

## Conflict of Interest

The authors declare that the research was conducted in the absence of any commercial or financial relationships that could be construed as a potential conflict of interest.

## Publisher’s Note

All claims expressed in this article are solely those of the authors and do not necessarily represent those of their affiliated organizations, or those of the publisher, the editors and the reviewers. Any product that may be evaluated in this article, or claim that may be made by its manufacturer, is not guaranteed or endorsed by the publisher.

## Footnotes

1
http://www0.cs.ucl.ac.uk/staff/g.ridgway/vbm/get_totals.m

2
https://stats.idre.ucla.edu/other/gpower/
